# The Impact of the COVID-19 Pandemic on Mental Disorders. A Critical Review

**DOI:** 10.3390/ijerph181910041

**Published:** 2021-09-24

**Authors:** Vicente Javier Clemente-Suárez, Marina Begoña Martínez-González, Juan Camilo Benitez-Agudelo, Eduardo Navarro-Jiménez, Ana Isabel Beltran-Velasco, Pablo Ruisoto, Esperanza Diaz Arroyo, Carmen Cecilia Laborde-Cárdenas, Jose Francisco Tornero-Aguilera

**Affiliations:** 1 Faculty of Sports Sciences, Universidad Europea de Madrid, 28670 Madrid, Spain; josefrancisco.tornero@universidadeuropea.es; 2 Grupo de Investigación en Cultura, Educación y Sociedad, Universidad de la Costa, Barranquilla 080002, Colombia; 3 Studies Centre in Applied Combat (CESCA), 45007 Toledo, Spain; 4 Departamento de Ciencias Sociales, Facultad de Ciencias Sociales y Humanas, Universidad de la Costa, Barranquilla 080002, Colombia; mmartine21@cuc.edu.co (M.B.M.-G.); jbenitez@cuc.edu.co (J.C.B.-A.); 5 Facultad de Ciencias de la Salud, Universidad Libre, Barranquilla 080005, Colombia; eduardoi.navarroj@unilibre.edu.co or; 6 Education Department, Universidad Antonio de Nebrija, 28240 Madrid, Spain; abeltranv@nebrija.es; 7 Department of Health Sciences, Public University of Navarre, 31006 Pamplona, Spain; pablo.ruisoto@unavarra.es; 8 Departamento de Salud, Universidad de la Costa, Barranquilla 000802, Colombia; ediaz5@cuc.edu.co; 9 Vicerrectoría de Investigación e Innovación, Universidad Simón Bolívar, Barranquilla 080005, Colombia; cacelaca6@gmail.com

**Keywords:** COVID-19, anxiety, depression, burnout, post-traumatic stress disorder, eating disorder, violence, apps

## Abstract

The COVID-19 pandemic has impacted the lives of the worldwide population. Citizens suffer the social, economic, physiological, and psychological effects of this pandemic. Primary sources, scientific articles, and secondary bibliographic indexes, databases, and web pages were used for a consensus critical review. The method was a narrative review of the available literature to summarize the existing literature addressing mental health concerns and stressors related to the COVID-19 pandemic. The main search engines used in the present research were PubMed, SciELO, and Google Scholar. We found the pandemic has had a direct impact on psychopathologies such as anxiety, increasing its ratios, and depression. Other syndromes such as burnout and post-traumatic stress disorder have increased with the pandemic, showing a larger incidence among medical personnel. Moreover, eating disorders and violence have also increased. Public authorities must prepare healthcare systems for increasing incidences of mental pathologies. Mental health apps are one of the tools that can be used to reach the general population.

## 1. Introduction

Originating in Wuhan (Hubei, China) on 31 December 2019, a total of 27 cases of pneumonia of unknown etiology led to a global viral pandemic (SARS-CoV-2) [1]. The exponential and global increase in the rate of infections and the first deaths were the triggers for the World Health Organization (WHO) to declare a pandemic on 11 March 2020. It has affected the population worldwide, with more than 110 million confirmed cases and more than 2.5 million deaths [2] at its peak. As of September 2021, this epidemiological crisis continues with about 219 million accumulated cases and 4.55 million accumulated deaths. Yet, around 5760 million vaccine doses have been administered and 2370 million citizens have been immunized, or 30.7% of the worldwide population [3]. The coming years will be marked in history as the era of COVID-19.

To date, data suggest an increase in the incidence of cumulative cases and deaths with a downward trend concerning local transmission. However, the slow process of administration of vaccines, and considering that 70% to 85% [4] of the world population must be vaccinated to obtain group immunity, means that the pandemic, its ravages, and restrictions are here to stay at least till the third quarter of 2023 [5]. To keep it in check and maintain the current downward trend in accumulated cases, serious control and containment measures are mandatory, which have led to immediate and serious concerns about the mental health of general society [6], with calls for urgent and direct actions [7]. If we look at other past epidemics such as severe acute respiratory syndrome (SARS) or the 2014 outbreak of Ebola, we can see that the extraordinary measures of containment, isolation, and social distancing undoubtedly lead to alterations in mental health [8], increasing perceived anxiety, depression, disturbing sleep, and quality of life [9]. However, the social and economic impact of COVID-19 has been greater than that of previous global pandemics. Authors point out that COVID-19 cost more in 2020 than the world’s combined natural disasters in any of the past 20 years [10]. The period of home isolation has been the longest in history [11], and the impact on macro- and microeconomics is comparable to that of the Great Depression of 1929 [12]. Sudden and abrupt changes in the lifestyle of citizens have increased domestic violence [13], drug abuse [14], reduction of physical activity [15], worsening of eating habits [16], and more passive and sedentary lifestyles [17]. All these things are risk factors for mental health [6].

Indeed, authors suggest that 1 in 7 US adults reported psychological distress back in April 2020, at the very peak of contagiousness [18]. From a survey of 190 million US citizens, authors found that appointments seeking help for mental health conditions, suicide attempts, drug and opioid overdoses, intimate partner violence, and child abuse and neglect were higher in mid-March through October 2020 during the COVID-19 pandemic, compared with the same period in 2019 [19]. This suggests that emergency care-seeking shifts during a pandemic, underscoring the need to integrate mental health, substance use, and violence screening and prevention services into response activities during public health crises [19]. Yet, there are two major population foci when studying the psychological impact of confinement and the pandemic’s consequences: the general population, and health-related professionals. Among health-related professionals, the ratios of emotional distress, anxiety, and depression are even higher than in the average population [20]. It is necessary to address and understand burnout syndrome and post-traumatic stress disorder as a potential problem, especially among doctors, nurses, and physicians, since they usually work with a high level of occupational exposure, with long working hours, as well as a high level of demand and task overload [21].

It is essential to attend to the processes of stress somatization and depressive episodes. Chronically maintained stress, anxiety, and depression will expose the subject to burnout syndrome [21], especially among medical workers [22,23]. Likewise, seeing that the current pandemic situation, as well as its restrictions, will remain until the third quarter of 2023, the risk of suffering PTSD in both the general and medical population is very high [24]. Thus, the current narrative review was designed to summarize the existing literature addressing mental health concerns and stressors related to the COVID-19 pandemic.

### Search Methods and Strategies for Research Identification

The protocol used consisted of a literature search, using primary sources, including scientific articles and secondary sources such as bibliographic indexes, databases, and web pages. We used PubMed, Embase, SciELO, Science Direct Scopus, and Web of Science, employing MeSH-compliant keywords including COVID-19, Coronavirus 2019, SARS-CoV-2, 2019-nCoV, Mental Health, Mental Pathology, Psychology, Depression, Stress, Psychiatry. We used articles published from 1 February 2020 till 13 May 2021, although previous studies were included to explain some information in several points of the review. The following exclusion criteria were used: (i) studies with old data, i.e., data not relevant to COVID-19/pandemics); (ii) inappropriate topics, being those not pertinent to the main focus of the present narrative review; (iii) Ph.D. dissertations, conference proceedings, abstracts, unpublished studies, and books. We included all the articles that met the scientific methodological standards and had implications for any of the subsections of this article, mental health, and COVID-19. The information treatment was performed by all authors of the review. Finally, articles were discussed by the authors to write the present review. A final 175 papers were considered relevant to the search criteria and appropriate for assessing our research objective.

## 2. A New Context, New Stressors

The COVID-19 pandemic has produced some of the most significant changes in sociological terms that human beings have experienced so far in the 21st century [25]. The situation of uncertainty, ignorance, and chaos has exposed people to new stressors that demand they adopt coping mechanisms to avoid being overcome by the situation [26,27].

Different kinds of stressors influence people differently depending on personal coping abilities and interpersonal capabilities [26]. Among those with relevance to COVID-19, individual stressors include confinement, losing routine, confusion, uncertainty, fear of contagion, reduced concentration, diminished physical activity and sunlight exposure, sleep disorders, heavy use of digital media, variations in eating routines, and high consumption of COVID-19 information on news and media [26,28,29,30,31]. Of the main stressors associated with the pandemic, we will focus on the following: social isolation, dependence on technology, fear of contagion, sociological and cultural aspects of biosecurity patterns such as the use of masks, and employment and home working.

Concerning isolation measures, people in their different conditions have had to stop having contact with others. This situation has been particularly detrimental to older adults living in long-term care institutions [32]. They have experienced profound isolation and become prisoners in their bedrooms. Extreme loneliness creates a risk of poor health, anxiety, depression, and worsening dementia [32,33,34,35]. In young people, interpersonal relationships showed multiple fractures, and with them, the desperation to share with others again. Consequently, thousands of clandestine parties have taken place in many territories, either due to a lack of recognition among the public of the implications of contagion for fragile health systems, or due to the urgent need to overcome confinement and to be able to experience physical contact with family and friends [36,37,38,39,40]. 

Isolation measures have accelerated our dependence on technology as a means of relationship in all expressions of human socialization. For example, education that slowly incorporated the use of information and communication technologies was forced to completely migrate to the digital world, revealing the multiple deficiencies in access and infrastructure in many territories [41,42]. Additionally, it exposed the limited digital skills of teachers and caregivers at home who, confined, were forced to accompany children in a more committed way in their training and educational process [41,43]. Some parents oppose and reject online learning due to its shortcomings, young children’s limited self-regulation, and lack of time and professional skills to assist their children [44]. These conditions represented stress to millions of teachers and families, who have needed to adapt quickly to streaming platforms to maintain the training process remotely, synchronously, or asynchronously with their students. Students have displayed a wide range of symptoms of anxiety facing online learning and feelings of disappointment with this methodology [41,45]. 

However, the stress related to the immersion into the technology world has other manifestations. During COVID-19, information about the disease and advised measures have circulated through a diversity of channels. The constant use of social media to access information results in confusion and overconcern, increasing stress, anxiety, and depression due to the fear of contagion [46,47,48]. Many people have increased their online connection to social media before sleeping [49], and pandemic dreams reflect mental suffering and fear of contagion [50]; however, this fear has also had fatal outcomes because many people have stopped attending their medical check-ups or even seeking help after heart attacks due to fear of contagion [51,52].

Another scenario of uncertainty and inconsistency that clashes with our habits has been related to courtesy protocols, which have changed due to biosafety protocols previously exclusive to medical personnel [53]. From making decisions on how to greet people to deciding how to dress, COVID19 has generated uncertainty during surreal scenarios with masked people whose facial expressions we must imagine, pretend to recognize, and intuit. Face masks decrease emotion-recognition efficiency and perceptions of closeness. These effects may be worrisome when certain emotion recognition is fundamental [54]. The formation of impressions and, therefore, the generation of adaptive responses in the brain about whether a person is in danger today is challenged by policies and epidemiological measures that generate cognitive and emotional discomfort, to the point that they have created social movements opposing these types of measures in many countries [55]. The COVID-19 pandemic has been associated with reducing trust and trustworthiness [56]. The pandemic biosafety protocols disturb daily social activity, particularly at night when new social contacts are desired [36]. Furthermore, the experiences concerning face coverings during the pandemic indicate that the expectation of stigma due to not wearing a mask (even with a valid exemption) inhibits some people from leaving home to go about their daily routine. The sociocultural dimensions of face coverings must allow engagement with the moral concerns of inequality and exclusion, including the potential for disadvantageous outcomes for marginalized groups [57].

Another cause of stress in the face of threats during the COVID-19 pandemic is the lack of employment, fear of losing it, or the uncertainty of not getting it [58]. As a result, freelancers, out of desperation, agree to precarious employment contracts, especially freelancer women [59]. Many creative professionals have lost their jobs, and freelancers and events producers have been hit the hardest [60]. Increasing unemployment has been associated with suicides worldwide during the pandemic [61]. On the other hand, we have people who must work in new conditions, many of them in confinement. Working at home has been associated with a lack of psychological need for employees to work effectively and engage with their families, and consequently poorer health [62]. Thus, the worsening conditions of workers in the pandemic increase the intensification of the unequal labor situations that characterize society. While domestic workers are complimented as heroes in public speech, this symbolic recognition is not extended to monetary remuneration [63]. Other people have been forced to migrate due to the economic repercussions of the pandemic affecting daily life and opportunities for both migrants and locals [64]. 

Another important factor is the socioeconomic disparities that have a direct impact on the mental health of the population [35,53]. Understanding the ethology of socioeconomic health disparities could assist public health authorities in preventing the morbidity of socially disadvantaged individuals [24]. Social inequities have many health effects; one of these is a potential relationship to sleep disturbances [60]. Socioeconomic status is an important factor that contributes to social inequities. Socioeconomic status is a marker of living conditions and habits that influence health by way of different processes, including stress-related mechanisms [53,59]. Low socioeconomic status is linked with poor nutrition, a fact that has a direct impact on health, especially in a pandemic [27]. This situation was present before COVID-19 and was intensified because of this same pandemic. In this regard is important to highlight that anxiety and depression are linked with sleep disturbances [24,27], how COVID-19 affects anxiety and depression, and how COVID -19 increases socioeconomic disparities in vulnerable groups with anxiety and depression. There is no doubt that there is a pattern linking socioeconomic status, COVID-19, and sleep health [28,30,35].

## 3. Anxiety Incidence during the COVID-19 Pandemic

Since the beginning of the pandemic in 2020, there has been a significant increase in psychological disorders as a result of the health crisis. One year after the declaration of the COVID-19 pandemic, it is possible to confirm that a high percentage of the population suffers symptoms associated with anxiety disorders [65]. It is known that exceptional situations, such as isolation, produce by themselves an aggravation of psychological disorders that can range from minor to severe, having a negative impact on people’s lives [66], from sporadic anxiety symptoms to more significant symptoms such as insomnia, depression, or acute stress disorders. However, there are also other elements that have contributed to the increase of anxiety in the population during this time, such as the uncertainty about a disease that does not seem to be abating, the fear of contagion, and the rapid spread of the virus, which will generate negative thoughts, fear, and sadness [67].

A study conducted to analyze the psychological impact of the virus in the city where it started, Wuhan, indicates that more than 50% of the population presented symptoms related to anxiety and depression in different degrees, and more than 70% of the population presented symptoms of fear and worry and anticipatory anxiety [68]. Other studies carried out in different countries have confirmed the prevalence of disorders associated with anxiety. In Spain, more than 2000 people were analyzed during the period of confinement, showing a high incidence of emotions such as fear and physiological distress, difficulties or alterations in sleep patterns, and depressive symptomatology [69]. In Colombia, another study along the same lines with participants aged between 18 and 70 years showed that more than 30% of the general population presented symptoms of excessive preoccupation and fear [70]. A study conducted in the first months of the pandemic in the USA revealed that firearm sales increased by 85% compared to the same month of the previous year. What is dangerous about this data is the direct relationship between firearm ownership and the risk of suicide. This risk becomes even higher if factors such as job loss, helplessness in the face of illness, or the loss of a family member are combined [71,72].

It is also necessary to attend to the aggravation of anxiety disorders already existing before the pandemic. In this scenario, these disorders have increased in prevalence and in many cases, medical attention is essential. This care has been reduced by the health crisis, leaving these patients without adequate clinical care, which exacerbates the situation [73]. It is clear that, in exceptional and novel circumstances, people tend to present symptoms associated with anxiety. In previous pandemics, such as the Spanish flu and HIV outbreaks, anticipatory anxiety resulting from the perception of imminent danger or threat and the risk of death appeared along with symptoms such as uneasiness, fear, and insecurity [74,75]. Associated with these symptoms we found negative, obsessive thoughts and even phobias. All this symptomatology will disappear only when the person can perceive the disease as something manageable and with no death risk [76]. 

Since control of the pandemic can only be possible with an effective vaccination that allows reaching herd immunity, it is a priority to maintain health measures and restrictions on mobility and reunion until this happens [77,78]. These measures involve the perception of insecurity that will keep the alert systems of the organism active; it is probable that the anxiety disorders detected during the pandemic will continue to the end of the pandemic.

## 4. Depression Incidence during the COVID-19 Pandemic

Depression is a psychiatric disorder characterized by feelings of sadness, loss, anger, or frustration, in which affected individuals tend to think negatively about the past, the world, and the future [79]. In the COVID-19 pandemic, depressive symptomatology has been studied as a recurrent manifestation of patients facing COVID-19; that is, how in previous studies, psychiatric morbidities have been shown in people with prior coronavirus infection to range from 10% to 35% in the post-disease stage [80,81].

These psychiatric consequences appear to be associated with the immune response due to the cytokine storm involved in the response to coronaviruses. This precipitates neuroinflammation, alteration of the blood–brain barrier, invasion of peripheral immune cells in the central nervous system (CNS), deterioration of neurotransmission, adrenal hypothalamic-pituitary axis (HPA), activation of microglia, and induction of indoleamine 2.3-dioxygenase (IDO), all pathways of interaction between immune systems and psychopathological mechanisms that support psychiatric disorders such as depression [80,81]. 

Similarly, psychological comorbidities exist among people who are exposed to life-threatening situations such as illness or isolation. In these conditions, feelings about being trapped, restricted access to outer space, not being able to go to work, the economic deficit, alterations in routines, separation of family and friends, having a person at risk in the family, scarcity of daily needs, reduction of wages, social isolation, and closure of educational institutions are risk factors that have been associated with the development of depressive symptomatology in the COVID-19 pandemic [79,82,83,84]. Likewise, belonging to socioeconomic groups with lower incomes and having little in savings is associated with being 1.5 times more likely to have symptoms of depression [85]. Similarly, it has been found that in countries where rigorousness rates are higher, depression levels tend to be higher [86].

Currently, the prevalence of depression reported in the general population due to the COVID-19 pandemic ranges from 14.3–24.3% in different studies conducted [82,87,88,89,90]. Symptoms of depression associated with COVID-19 are more common in women, people with low [87,91] socioeconomic status, students, and health workers [89,92], with the latter group reporting a prevalence ranging from 2.3–46% due to several factors, including workload [93,94,95,96].

As for the manifestation of this symptomatology, it has been found according to lifecycle studies that children are most likely to develop attachment and fear related to the concern that family members may contract the disease, as well as [97] opposition-challenging behaviors [98]. Younger patients tend to have higher levels of depression, associated with deprivation of liberty and the closure of schools [99,100], while older adults have recorded depression related to decreased activity level, sleep quality, well-being, and cognitive functioning [101], in addition to those who are widowers or separated being associated with a higher risk of developing emotional disorders during the COVID-19 pandemic [102].

Therein lies the importance of generating follow-up studies with these patients, both before and after infection, to estimate the burden on mental health due to deficits in health behaviors associated with difficulty sleeping, reduction in physical activity [79,87], and increased consumption of psychoactive substances [79]. These are challenges that health systems will face globally in the aftermath of the coronavirus pandemic in the general population, related to a new, possible mental health-related pandemic. 

This is why it is vital for the general population to follow the recommendations of the experts to take care of their mental health, avoiding [82] unscientific information about COVID-19, maintaining regular exercise routines [87], and communicating with family members [79] among other daily routines for mental health, such as healthy eating, sleep, socialization, leisure activities, work, and study. At the public health level, it is important to consider that uncompromising and strict policies have been associated with higher rates of depression in the general population; therefore, it is important that in establishing policies on issues such as social distancing and quarantine, each country examines its context not only regarding epidemiological conditions but also the mental health conditions of its population [86], seeking to find solutions that contribute to all the areas in which this disease causes involvement.

## 5. Burnout Incidence during the COVID-19 Pandemic

Burnout (BO) is defined by the World Health Organization (WHO) as a syndrome resulting from chronic workplace stress that has not been successfully managed [103], characterized by energy depletion and mental distancing from one’s job, producing feelings of negativism and cynicism, and reduced professional [104,105] efficacy, and associated with higher rates of substance abuse, depression, and suicide [104,105,106].

Over the past 10 years, BO has become a significant psychosocial problem [107]. Healthcare professionals as a group are particularly susceptible, mainly due to the demanding nature of their profession and work environment [104,105]. Thus, the prevalence of BO symptoms such as anxiety, depression, lower satisfaction, and PTSD are higher in this professional group than in other professions [108]. Studies conducted at the beginning of the pandemic in China suggested that the frequency of BO is significantly less in frontline workers than in healthcare professionals working on their usual ward. The conclusion was that directly addressing the virus on the frontline is thought to bring a greater sense of situation control, considered to be a leading motivation for engagement that decreases chances of BO occurrence [109].

Yet, COVID-19 has exacerbated stressors in healthcare systems where physicians’ burnout response to workplace stress is already epidemic [110]. In this line of thought, the pandemic presents a sort of perfect storm regarding the intersection of chronic workplace stress and the acute traumatic stress imposed by the pandemic [111]. Authors described eight specific sources of COVID-19-related physician anxiety: access to appropriate personal protective equipment, exposure to COVID-19 at work and taking the infection home to their family, not having rapid access to testing if they develop COVID-19 symptoms and concomitant fear of propagating infection at work, uncertainty that their organization will support/take care of their personal and family needs if they develop an infection, access to childcare during increased work hours and school closures, lack of support for other personal and family needs as work hours and demands increase (food, hydration, lodging, transportation), being able to provide competent medical care if deployed to a new area (e.g., non-ICU nurses having to function as ICU nurses), and lack of access to up-to-date information and communication [112]. These sources of stress and anxiety fall outside the regular work experience and are drivers of both BO and PTSD [111].

Most of the literature concerning BO syndrome and its relationship with the COVID-19 pandemic are centered on healthcare professionals as they are the most affected by this situation. However, the psychological effects produced by the current situation also extend to the general population, as shown by a study conducted in Italy during the first wave of the pandemic. A third of the study’s sample presented psychiatric symptoms of stress, anxiety, and depression during the first month of the COVID-19 pandemic outbreak; more than 50% of the subjects presented sleep disturbances and 13% appeared at risk of developing PTSD. Furthermore, younger age and female gender appeared to be risk factors for the development of psychiatric symptoms [113].

Burnout has also developed among parents during the COVID-19 pandemic [114]. In normal situations, parents already experience stress related to their role as parents [115]. For most parents, parent-related stress is transitory but for 5–20% this stress can escalate to the level of parental burnout [116], similar to the standard BO syndrome with the same symptoms as previously described [104,105]. Some conditions predictive of parental burnout that are currently more common during the COVID-19 pandemic are unemployment, financial insecurity, low levels of social support from family and friends, and a lack of leisure time [117,118,119], conditions that can also be extrapolated to the general population.

In order to mitigate BO, systems-based interventions should be implemented [120]. Individual practices focus on managing the emotional aspects of stress and fear and leverage positive psychology, mindfulness practices, and embodiment to combat the fight-or-flight response, as well as symptoms of emotional exhaustion and depersonalization [121]. While systems-based interventions should focus on creating a work environment that promotes the development of individual practices, department resources should be directed toward creating a physically safe work environment and support the development of an infrastructure that allows physicians to work from home [111]. 

## 6. Post-Traumatic Stress Disorder Incidence during the COVID-19 Pandemic

Most of the population is robust in the aftermath of tragedies and does not succumb to psychopathology. Nonetheless, post-traumatic stress disorder (PTSD) caused by trauma is a major concern in “traditional” natural disasters, technological catastrophes, and intentional acts of mass destruction. Medical illnesses resulting from natural causes, such as a life-threatening viral infection, may not match the current trauma criteria for a diagnosis of PTSD, but they may lead to other psychopathologies, such as depression and anxiety disorders [75]. However, according to the DSM-5 [122], PTSD is a stressor-type stimulus that may induce intense feelings of threat to life and physical integrity, and intense fear, helplessness, or horror. Thus, by definition, COVID-19 would meet the definition of a traumatic event. In this line, there are several stimuli that can make the subject suffer PTSD due to COVID-19, among them: subjects who have suffered serious COVID-19 symptomatology and even experienced potential risk of death; subjects who have experienced the loss of family members, close friends, and relatives, witnessing suffering and death of other and the inability of grieving; individuals who have closely experienced and been chronically exposed in the front line to the virus and its ravages (e.g., journalists, first responders, medical examiners, and hospital personnel) [123].

A retrospective look at previously experienced global pandemics suggests that mental health symptoms and disorders are likely to arise among the population, such as anxiety, depression, insomnia, and PTSD. According to a systematic review of past pandemics, including SARS, MERS, and the current COVID-19, 14% to 61% of infected individuals experienced serious psychiatric and neuropsychiatric problems) during the illness, and 14.8% to 76.9% continued with these problems once they had overcome the disease. These values vary depending on the world zone, and thus the sociocultural, economic, health, and political characteristics of the country where the study was carried out [124]. Another comprehensive study found a significant prevalence of anxiety, sadness, post-traumatic stress disorder, and psychological distress symptoms among COVID-19-affected populations in several nations [74]. Furthermore, according to a recent meta-analysis, worldwide the pooled prevalence of depression is 15.97%, anxiety 15.15%, insomnia 23.87%, PTSD 21.94%, and psychological distress 13.29%, while gender, geographical location, and occupation as a healthcare worker did not create any significant differences (except for insomnia, which was more prevalent among healthcare workers).

However, the appearance of PTSD can differ greatly depending on the subject, their psychometric profile, lived experience, and the degree of exposure. Indeed, some people may be more sensitive to the emotional impacts of pandemics than others. There are groups at greater danger of contagion and risk of death (such as the elderly [125], those with compromised immune systems [126], and those who live or receive care in congregate settings [75]). Groups with preexisting psychiatric and medical conditions also have a higher risk of negative psychosocial outcomes [127]. Front line personnel, chronically exposed to the virus, are emotionally and ethically confronted with making decisions about the lives of other people given the health collapse [128]. Groups of the adolescent and pre-adolescent population have seen their day-to-day, habits and face-to-face education interrupted [129]. These and other population groups, due to their higher incidence and risk of suffering from PTSD and negative psychosocial outcomes, should be the focus of prevention measures in terms of mental health, psychoeducation, and psychosocial assistance, as suggested by previous authors.

Early monitoring and care are essential in this regard. The symptoms caused by the presence of PTSD can be seen in three aspects: a sensation of emotional numbness, depersonalization, and arousal symptoms (difficulty sleeping, irritability or quickly agitated, difficulty focusing) [111]. However, the symptoms may not be evident for at least one month following a stressful incident, or even years afterward, a fact which limits the diagnosis. The diagnosis given in the first month following a traumatic incident is acute stress disorder, which is associated with a sense of intrusion, dissociation, bad mood, avoidance, and arousal symptoms. After a stressful occurrence, the incidence of acute stress disorder ranges from 5% to 20%. Importantly, intervention in this early phase can reduce the progression to PTSD [130].

## 7. Eating Disorders Incidence during the COVID-19 Pandemic

Recent research suggests COVID-19 is not just a pandemic, but a ‘syndemic’. This term highlights the aggregation of the severe acute respiratory syndrome coronavirus (SARS-CoV-2) and non-communicable diseases clustered within social groups following a socioeconomic gradient [131]. The syndemic nature of the pandemic raises concerns about other socially related problems such as eating disorders. In fact, recent studies suggest that COVID-19 may precipitate the development of eating disorder (ED) behaviors among some and exacerbate existing pathology among others. 

According to a recent study, there are three ways in which COVID-19 may negatively affect the risk of eating disorders [132]. 

First, by changes in daily-life routines, which include constraints on outdoor physical activities and therefore, potential increased concerns about weight and shape, and a negative impact on eating, exercise, and sleeping patterns, which may in turn increase ED risk and symptoms. Relatedly, the pandemic and accompanying social restrictions may deprive individuals of social support and adaptive coping strategies, thereby potentially elevating ED risk and symptoms by removing protective factors [133]. 

On one hand, in the context of the COVID-19 pandemic, restrictions on daily activities and movements, particularly in urban areas, have been the norm. These measures include work and study from home and prevention of all non-essential activities, negatively affecting both eating and physical activity, and therefore, increasing the risk of EDs. For example, the absence of clear routines and markers of time and space, and stocking up on more food than usual, may lead to increased time being around food and snacking, increasing the risk of binge eating and other EDs. Additional limitations in access to regular physical activity may lead to changes in body shape and body weight, resulting in concerns and disordered eating [132,133].

Social distancing measures and confinement measures result in a barrier to social support, a well-known protective factor when people are exposed to stress, and prevent access to face-to-face care in cases where needed. Instead, the risk of coping or emotional regulation strategies such as emotionally driven eating or restrictive eating may occur [133].

Second, social distancing also increases social media use as a means of communication, resulting in increased exposure to video conferring and also (social) media, and therefore increased exposure to thin-ideal- and diet culture-related content may increase ED risk and symptoms. For example, a common feature of body image and eating concerns is body image avoidance (do not look in the mirror); so, video conferring may result in an excessive focus on face and appearance, which may increase the risk of EDs. Previous studies have shown that exposure to media coverage of stressful and traumatic world events was associated with an increased risk of eating disorders [132]. 

Third, COVID-19 may increase health concerns, and those emotional fears associated with the risk of contamination may result in a reduction in the purchase or selection of food. Indeed, in EDs, health concerns are associated with the manipulation of diet, for example in anorexia nervosa. For example, some rigid and restrictive diets are believed to have immunity-related health benefits; however, they lead to increased risk of COVID-19 [132].

Finally, restrictions and social distancing measures in response to COVID-19 have resulted in a general increase in stress and negative affect due to the health issues and increased social isolation or loneliness that are core risk factors for EDs, such as binge eating, purging, or restrictive patterns. For example, emotional eating under stress usually focuses on high-carbohydrate foods, which can result in binge eating. However, more studies are needed to compare population-representative samples to provide evidence about the impacts of COVID-19 and their associated measures on the risk of EDs.

## 8. Violence Incidence during the COVID-19 Pandemic

One year after the COVID-19 pandemic was declared by the WHO [134], a growing number of studies about increased violence—mainly against women and children—have been published from the first months of 2020 to date. In the following tables, the results of research carried out in low- and middle-income countries that identify trends, analyses of risk factors, and the impact of violence prevention programs will be analyzed.

Most of the articles evidenced an increase in violence (an increase of up to 40% depending on the country and type of study) when comparing the pre-pandemic and pandemic periods. Given the large percentage margin, in Table 1, a detailed breakdown is made by authors, country, and outcomes. 

Yet, regardless of the percentage increase, authors agree that an increase in violence was more accentuated during national and local lockdowns. Regarding women, the predictors most frequently associated with the increase of violence were being married, being unemployed (which is accentuated by the lack of economic independence of women), different nationality to the husband, living in rural areas, being a married minor, or less than 16 years of age. Both physical and psychological violence is reported in most of these studies. Data collection methods were mostly based on digital surveys (e.g., Google forms) which may suggest underreporting, as answering surveys depends on the abused person having access to a cellphone or other device (i.e., they will regularly not have access) (Table 1).

The results of research evaluating risk factors, describing experiences of violence, or examining prevention programs during the pandemic will be described below. Of the studies that documented important risk factors for increased violence, several of those that stood out were being married, being unemployed (either for the victim or the perpetrator), having lost family income due to the pandemic, and tendencies of the perpetrator’s substance abuse. Of particular interest are studies from a variety of countries that evidenced increased economic vulnerability, whether in the form of unemployment, reduced family income, or food insecurity, linked to an increased risk of violence. New dynamics have expanded beyond lockdowns including widespread economic crises, and effects on violence against women (including IPV) and children. These will continue to evolve and are likely to outlast the health effects of COVID-19 and lockdown measures.

Few studies point at potential protective factors such as evidence from India [146] and Ethiopia [150], which suggests that higher education (for both the victim and the perpetrator) decreases the risk of violence. Levels of intimate partner violence (IPV) were twice as high for illiterate women in Ethiopia as for women who had completed high school. In India, rates of IPV were significantly lower for husbands or wives who had a higher education/professional degree. Employment status can also be a protective factor. Studies in Jordan [135] and Iran [140] showed significantly lower rates of violence for employed women, reflecting the association between losing employment or income during the pandemic with an increase in violence against women or children.

Two studies focused on physical violence against health workers, specifically Iranian nurses [151], and mental illness in Chinese health workers who experienced aggression in their workplace [152]. Two studies specifically focused on pregnant women in Iran [153] and Ethiopia [154], documenting high rates of violence during the pandemic. Only one study included an evaluation of a violence prevention or mitigation program. The program, a youth empowerment program in Bolivia, found a decrease in violence towards girls of almost ten percentage points (or 46% in relation to the control group), seven months after its completion [155] (Table 2).

Government authorities and women’s rights organizations should work together towards improving IPV prevention and widespread violence against women. An effective prevention strategy must emphasize recognizing and acknowledging the magnitude of the problem, enhancing awareness of the problem and leading resources to address it, and ensuring social and economic stability. The lessons learned about the increased prevalence of IPV and widespread violence against women during the COVID-19 pandemic and the need to adopt appropriate strategies to prevent and address it will be valuable for future similar crises.

## 9. Use of Mental Health Apps during the COVID-19 Pandemic

The COVID-19 has played a significant triggering role, aggravating a wide range of mental disorders [75]. However, the COVID-19 pandemic has also accelerated the development of mental health apps, currently reaching a number between 10,000 and 20,000 according to One Mind PsyberGuide, which might help to identify and track mental problems. The specific number of mental health apps is difficult to estimate because new apps are constantly being developed and older ones are removed from the market. Unfortunately, most apps are developed by small teams without plans for long-term support and their utility is questionable.

In a recent study, it was found that just two apps, Headspace and Calm, accounted for 90% of monthly active users [161]. People do not generally use these mindfulness apps as a replacement for therapy but as additional support to enhance progress outside the therapy office.

The Food and Drug Administration regulates the so-called “digital therapeutics” that aim to provide actual treatment, but it does not regulate self-help apps that fall in the broad category of wellness and, in some cases, apps might be misleading. For example, the content of currently available apps for bipolar disorder is not in line with practice guidelines or established self-management principles [162].

Regarding the COVID-19 pandemic, app-based contact tracing started to be used. The epidemiological evidence shows that app-based contact tracing can suppress the spread of COVID-19 if a high enough proportion of the population uses the app, and that it can still reduce the number of infections if uptake is moderate. The available evidence suggests that app-based contact tracing may be a viable approach to control the diffusion of COVID-19 [163].

In this line, the COVID-19 Symptom Tracker is an app-based daily self-reporting tool also used in the pandemic. Self-reported symptom tracking helps to identify novel symptoms of COVID-19 and to estimate the predictive value of certain symptoms. This aids in the development of reliable screening tools. Clinical screening with a high pretest probability allows for the rapid identification of infections and the cost-effective use of testing resources. Based on the results obtained by this app, researchers suggested that loss of smell and taste be considered cardinal symptoms; and that diabetes is a risk factor for a highly symptomatic course of COVID-19 infection [164].

## 10. Discussion, Highlights, and Practical Applications

Mental health issues should be considered as the second pandemic. Authors suggest that the current pandemic situation, as well as its restrictions, will remain until the third quarter of 2023 [165]. Thus, the risk of suffering several mental pathologies in both the general and medical population is very high, as well as increasing social inequities [166]. Indeed, almost one year after COVID-19 began, a high percentage of the population suffers symptoms associated with anxiety and depression disorders [167,168]. Yet, symptomatology will disappear only when the person can perceive the disease as something manageable and with no death risk. Strategies and policies such as social distancing and quarantine, and limitations in mobility, will need to be examined by each country regarding not only its epidemiological context, but also the mental health conditions of its population [169]. A greater number of state and government questionnaires to track these signs would be ideal to help make decisions regarding the health of the citizenry.

Regarding burnout, especially acute in medical personnel, systems-based interventions should be implemented. Individual practices should focus on managing the emotional aspects of stress and fear and leveraging positive psychology, mindfulness practices, and embodiment to combat the fight-or-flight response as well as symptoms of emotional exhaustion and depersonalization. Likewise, there should be a similar focus regarding PTSD given its high incidence among COVID-19 sufferers, depending on the country and population group (14% to 61% of infected subjects, and 14.8% to 76.9% once they have overcome the disease). Special vigilance is required from the institutions involved in early care, since intervention in the early phase can reduce the progression to PTSD. Thus, a key factor is identification of emotional numbness or depersonalization and arousal symptoms such as difficulty sleeping, irritability or becoming quickly agitated, and difficulty focusing [167]. 

These symptoms and mental health problems are aggravated by inappropriate lifestyles. The quarantine has highlighted changes in lifestyles, especially nutritional ones, which have been shown to have a direct impact on mental health [167,169]. Thus, restrictions and social distancing measures in response to COVID-19 have resulted in a general increase in stress and negative affect due to the health issues and increased social isolation or loneliness, which are core risk factors for eating disorders such as binge eating, purging, or restrictive patterns. Indeed, emotional eating under stress usually focuses on high-carbohydrate foods, which can result in binge eating [170,171,172,173]. Thus, psychiatrists and mental health professional should also focus on assessing nutritional habits of their patients as part of their daily routine.

Lastly, the increase in violence (an increase of up to 40% reported depending on the country and type of study) when comparing the pre-pandemic and pandemic periods), especially IVP towards women during the COVID-19 pandemic, highlights that the need to adopt gender-based strategies to prevent and address it will be valuable for future similar crises. 

It is highly encouraged that there be training and dissemination among psychiatric, psychological, medical personnel and the general population regarding the use of platforms and applications such as the One Mind PsyberGuide, which can serve as the main focus of primary care when identifying and quantifying symptoms and incidence [174,175].

## 11. Conclusions

The COVID-19 pandemic is a new contextual stressor that has negatively affected the incidence of anxiety, depression, burnout, post-traumatic stress disorder, eating disorders, and violence. Public authorities must prepare healthcare systems for the increasing incidence of mental pathologies, with mental health apps being one of the tools available to reach the general population.

## Figures and Tables

**Table 1:** Characteristics of violence studies.

Author	Country	Data Source	Analyzed Period of Time	Outcomes
Abuhammad 2020 [135]	Jordan	Online survey	May 2020–July 2020	Increase in intimate partner violence (IPV) (40%). Only 10% of abused women had been abused before the quarantine.
Aolymat 2021 [136]	Jordan	Online survey	September 2020	Increase in IPV (20.5%). Difficulties when accessing sexual and reproductive health services (increase from 35% to 41% when comparing pre- and post-pandemic periods)
Berniell and Facchini 2020 [137]	Argentina; Brazil; Chile; Colombia; France; Germany, Italy; Mexico; United Kingdom; United States.	Google search data; Google mobility data	March to October 2020	31% increase in domestic violence against women.
Dai et al., 2021 [138]	Hubei, China	Police records	January 2019 to June 2020	During the pre-pandemic period, 3.9% of all calls were related to IPV. During confinement, that percentage increased to 14.8% and 6.9%.
Fabri et al. [139]	Nigeria; Mongolia; Suriname	Face-to-face survey.	Multiple indicator cluster surveys (MICS) of Nigeria (2016), Mongolia (2018), and Suriname (2018). Data collected from mothers or other caregivers during the pandemic.	Findings evidence that the models predict large increases (35% to 46%) in violent discipline scores in “high restriction” scenarios and smaller increases (4% to 6%) in “low restriction” scenario scores.
Fereidooni et al., 2021 [140]	Isfahan, Iran	Survey data (face-to-face before the pandemic and by phone during the pandemic)	Pre-pandemic and post-pandemic	The prevalence of IPV (Intimate Partner Violence) during COVID-19 has increased from 54% (pre-pandemic) to 65 % (post-pandemic). More than 25% of women reported the first incidence of IPV during COVID-19. The participation of women in paid employment decreases the probability of exposure to IPV.
Guglielmi et al., 2020 [141]	Bangladesh (Rohingya and Bangladeshi teens)	Telephone surveys of adolescents aged 10–14 and 15–19 (1761), qualitative interviews with adolescents aged 15–19 (30), and interviews with key informants (7)	March 2020 to August 2020	8% of the adolescents surveyed (boys and girls) reported an increase in gender-based violence during the pandemic. Around a third of boys and a fifth of girls living in the camps reported an escalation of violence by the police and military force to impose containment measures. Married girls were twice as likely as single girls to report an increase in gender-based violence in the community. The pandemic has led to a decline in the reported health status of Rohingya adolescents, exacerbating food insecurity, educational and economic marginalization, and risks to physical integrity, both among girls and boys.
Mahmood et al., 2021 [142]	Kurdistan region, Iraq	Self-completed online questionnaire after COVID-19 confinement periods	June 2020	Significant increases in violence were observed from the period before confinement to the period of confinement for any type of violence (32.1% to 38.7%), emotional abuse (29.5% to 35.0%), and physical violence (12.7% to 17.6%). Regarding emotional abuse, significant increases were observed in humiliation (24.6% to 28.3%) and intimidation (14.2% to 21.4) during confinement. Concerning physical violence, significant increases were observed in arm twisting or hair pulling (9.0% to 13.0%) and hitting (5.2% to 9.2%) during confinement. Forcing to have sex also increased significantly during confinement (6.6% to 9.5%).
Pattojoshi et al., 2020 [143]	India	Self-completed online questionnaire	May 2020	The study reports an IPV rate of 18.1%, of which verbal and emotional violence was the most common, followed by physical and sexual violence. Approximately 5% of women reported experiencing violence for the first time since confinement began, and of those who reported experiencing it before, 78% reported an increase since confinement. The most commonly perceived reasons for violence were: unemployment, financial limitations, inability to socialize, staying at home (husband-forced), and sharing of childcare responsibilities.
Pinchoff et al., 2021 [144]	Nairobi, Kenya	Phone interviews	April to June of 2020	A survey conducted in informal settlements. Results reported increases in violence against women inside (IPV) and outside the home (45% and 24%, respectively). 8% of women are more likely to report a higher risk of IPV (compared to men), particularly in households with greater food insecurity.
UNFPA et al., 2021 [145]	Bangladesh, India, Indonesia, Malaysia, Nepal, Thailand, Philippines, Singapore	Social media data; internet search data; big data	October 2019 to September 2020	Online misogyny increased during the lockdown in all countries examined. Online support and services for survivors increased as well. Online help-seeking increased by 10–70% in most countries.
Sharma and Khokhar 2021 [146]	India	Online survey	April 2020	Approximately 7.4% had faced IPV during the confinement. 85.7% of people who faced IPV reported a higher frequency of IPV during confinement. 57% of the victims chose to ignore the situation or used meditation techniques to cope with the situation.
Egger et al., 2021 [147]	9 countries in Africa (Burkina Faso, Ghana, Kenya, Rwanda, Sierra Leone), 3 in Asia (Bangladesh, Nepal, Philippines), and one in Latin America (Colombia)	Phone or cellphone survey	April 2020 to June 2020	Decreases in employment and income were evidenced in all settings since March 2020. The proportion of households experiencing a drop in income ranged from 8% to 87% (median, 68%). Coping strategies at home and government assistance were insufficient to maintain pre-crisis living standards, leading to widespread food insecurity and dire economic conditions even after three months of the crisis. Even in Colombia, the country in our sample with the highest GDP per capita and therefore potentially the greatest financial resources to deal with the crisis, the majority of respondents reported declines in income (87%) and employment (49%) and an increase in food insecurity (59%).
Venter et al., 2020 [148]	Johannesburg, Southe Africa	Review of medical records	June 2019 to June 2020	25% decrease in trauma due to interpersonal violence between 2019 and 2020. Decrease of 40% in secondary traumas and traffic accidents between 2019 and 2020.
Agüero (2021) [149]	Peru	Telephone records of the IVP helpline	April to July 2020.	Almost 60% of women had experienced violence before COVID-19. The incidence rate of calls increased by 48% between April and July 2020, with effects increasing over time. The increase in calls was found in all Peruvian states.

**Table 2:** Violence outcomes by country, data source and period time analyzed.

Author	Country	Data Source	Analyzed Period of Time	Outcomes
Ghanbari et al., 2020 [151]	Rasht, Iran	Self-completed online survey	August 2020	The prevalence of verbal abuse of nurses was 62.5% during the first 6 months of the pandemic and was generally perpetrated by patients or their families. The prevalence of physical violence was 17.8%.
Gulesci et al., 2021 [155]	Bolivia	Telephone interview	February 2021	Girls participating in a youth empowerment program in Bolivia were 9.6% less likely to report experiencing violence compared to girls in a control group.
Haddad et al., 2020 [156]	Lebanon	Online electronic survey	February 2021	Women receiving psychological violence during COVID-19 lockdown have a lower, but not significantly lower, probability of pregnancy and a higher probability of unwanted pregnancy.
Hajj et al., 2021 [157]	Lebanon	Online electronic survey	May 2020	IPV was significantly associated with increased stress and insomnia, was weakly associated with anxiety and well-being, and was not significantly associated with post-traumatic stress symptoms.
Krishnakumar and Verma 2021 [158]	India	Data were obtained from wide circulation newspapers in India	March 2020 to May 2020	The symbolic value associated with women by the perpetrators and the lower visibility and accessibility of the perpetrators made women suitable targets for IPV. Finally, the scarcity of police force and travel restrictions reported from formal and informal sources resulted in the absence of capable guardians. We concluded that changes in people’s routine activities during the COVID-19 lockdown provided more opportunities for IPV.
Mahapatro et al., 2021 [159]	India	Qualitative study (phone call)	January 2020 to May 2020	Surviving women found it much more difficult to access services and social support networks to cope with domestic abuse during the period of confinement.
Naghizadeh et al., 2021 [153]	Tabriz, Iran	Self-completed survey at the hospital	May 2020 to August 2020	More than a third of pregnant women (35.2%) suffered IPV. The most common type of violence experienced was emotional violence (32.8%), followed by sexual violence (12.4%), and physical violence (4.8%).
Rockowitz et al., 2020 [160]	Kenya	Personal interviews	March 2020 to November 2020	Children were more likely than adults to be attacked during the day, by a single perpetrator rather than multiple perpetrators, and in a private rather than public setting. Children were more frequently raped by neighbors and family members, while adults had the same probability of being attacked by strangers and acquaintances. On average, the children in the sample were four years younger compared to the median age reported in the national samples before the pandemic (12 years versus 16 years). Survivors were more likely to be female than male.
Tadesse et al., 2020 [150]	Ethiopia	Personal survey	June 2020 to July 2020	Approximately 22% of those surveyed experienced at least one form of IPV (physical, psychological, sexual) during confinement. The most important determinants of having experienced violence were being illiterate or having an illiterate husband, having a substance-using husband, and the community’s tolerance of violence.
Teshome et al., 2020 [154]	Ethiopia	Open data kit	August 2020 to November 2020	In the sample, the prevalence of IPV in pregnant women was 7.1%, and among them, 72% reported emotional violence, 49% reported sexual violence, and 30% reported physical violence. A significant predictor of IPV was having a husband who chewed khat and drank alcohol.
Wang et al., 2020 [152]	China	Online electronic survey	February 2020	Rates of medical violence at work were 20.4% during the COVID-19 outbreak, and those who had experienced workplace violence were more likely to have elevated mental health problems.

## Data Availability

All data used are in the manuscript.
